# Development and validation of nomogram to predict overall survival and disease-free survival after surgical resection in elderly patients with hepatocellular carcinoma

**DOI:** 10.3389/fonc.2024.1395740

**Published:** 2024-05-24

**Authors:** Yuan Tian, Yaoqun Wang, Ningyuan Wen, Yixin Lin, Geng Liu, Bei Li

**Affiliations:** ^1^ Division of Biliary Surgery, Department of General Surgery, West China Hospital, Sichuan University, Chengdu, China; ^2^ Research Center for Biliary Diseases, West China Hospital, Sichuan University, Chengdu, China

**Keywords:** prognosis, recurrence, nomogram, hepatocellular carcinoma, elderly

## Abstract

**Background:**

Hepatocellular carcinoma (HCC) is one of the common causes of tumor death in elderly patients. However, there is a lack of individualized prognostic predictors for elderly patients with HCC after surgery.

**Method:**

We retrospectively analyzed HCC patients over 65 years old who underwent hepatectomy from 2015 to 2018, and randomly divided them into training cohort and validation cohort in a ratio of 3:1. Univariate Cox regression was used to screen the risk factors related to prognosis. Prognostic variables were further selected by least absolute shrinkage and selection operator regression model (LASSO) and multivariate Cox regression to identify the predictors of overall survival (OS) and disease-free survival (DFS). These indicators were then used to construct a predictive nomogram. The receiver operating characteristic curve (ROC curve), calibration curve, consistency index (C-index) and decision analysis curve (DCA) were used to test the predictive value of these independent prognostic indicators.

**Result:**

A total of 188 elderly HCC patients who underwent hepatectomy were enrolled in this study. The independent prognostic indicators of OS included albumin (ALB), cancer embolus, blood loss, viral hepatitis B, total bilirubin (TB), microvascular invasion, overweight, and major resection. The independent prognostic indicators of DFS included major resection, ALB, microvascular invasion, laparoscopic surgery, blood loss, TB, and pleural effusion. In the training cohort, the ROC curve showed that the predictive values of these indicators for OS and DFS were 0.827 and 0.739, respectively, while in the validation cohort, they were 0.798 and 0.694. The calibration curve nomogram exhibited good prediction for 1-year, 2-year, and 3-year OS and DFS. Moreover, the nomogram models exhibited superior performance compared to the T-staging suggested by C-index and DCA.

**Conclusion:**

The nomogram established in this study demonstrate commendable predictive efficacy for OS and DFS in elderly patients with HCC after hepatectomy.

Core Tip: The purpose of this retrospective study is to screen the risk factors of survival and recurrence in elderly patients with HCC after hepatectomy. The nomogram included cancer embolus, viral hepatitis B, overweight, major resection, ALB, microvascular invasion, laparoscopic surgery, blood loss, TB, and pleural effusion as predictors. The calibration curve of this nomogram was good, indicating credible predictive value and clinical feasibility.

## 1 Introduction

Primary hepatic cancer is a common malignant tumor of the digestive system, more than 90% of which is hepatocellular carcinoma (HCC). It is the sixth-highest incidence of malignant tumors in the world, accounting for 8.3% of cancer-related deaths worldwide, and is the third most frequent cause of cancer-related death (1). As one of the most populous countries with the largest number of hepatitis B patients in the world, the new cases of HCC in China account for 45% of the new cases in the world every year, and this proportion is also unceasingly growing (2). Therefore, HCC is still a major global health problem to be solved.

In recent years, the aging of the population has become a global problem, which has caused people’s concern that it will increase the morbidity of cancer. Clinical research has already confirmed that aging is an established risk factor for HCC (3). Research related to aging shows that cell and tissue aging caused by DNA damage, epigenetic changes, oxidative stress, and mitochondrial dysfunction will also increase the risk of malignant tumors (4). According to statistics, about 80% of HCC cases are elderly patients (5). Given the increase in life expectancy and the aging of the population around the world, it is expected that the number of elderly HCC patients may continue to rise.

At present, hepatectomy, liver transplantation, or radiofrequency ablation has become a recognized surgical treatment for HCC. As far as hepatectomy is concerned, the methods of surgery have developed from wedge-shaped resection or conventional hepatectomy to minimally invasive and precise hepatectomy (6). The concept of Enhanced Recovery After Surgery (ERAS) put forward in recent years emphasizes the integration of preoperative individualized disease assessment, surgical plan formulation, and perioperative rehabilitation treatment to minimize surgical trauma, protect remaining liver function, and strive for the best rehabilitation effect (7).

Although liver resection, the most commonly used surgical modality, has shown good results in elderly patients (8, 9), it is still a complex procedure (10). At the same time, due to the decline of physical function and underlying health conditions, elderly patients have a higher probability of severe postoperative complications, which leads to poor prognosis (11). Therefore, for such elderly patients, there may be large differences in prognosis due to individual conditions. However, there is no individualized prognostic prediction model for elderly HCC patients after liver resection.

This study aims to retrospectively analyze the relationship between perioperative indicators and tumor prognosis in elderly patients. Consequently, the objective is to propose an individualized prognosis prediction scheme for elderly HCC patients after surgery.

## 2 Materials and methods

### 2.1 Patient characteristics

We collected clinical and follow-up data of elderly patients > 65 years of age who underwent radical hepatectomy for HCC at West China Hospital of Sichuan University (Chengdu, Sichuan Province, China) from January 2015 to September 2018. The preoperative diagnosis of HCC was performed according to the criteria of the American Association for the Study of Liver Diseases (AASLD) (12). The absolute contraindications for liver resection are American Society of Anesthesiologists (ASA) grade >= III, ascites, extrahepatic metastasis, unresectable large vessel tumor invasion, or future residual liver <40%-50% (13). The inclusion criteria were as follows:(1) Patients aged >= 65 years (male or female); (2) Primary hepatocellular carcinoma was confirmed by postoperative pathological examination; (3) The years of diagnosis were 2015–2018; (4) Without the absolute contraindications for liver resection; (5) no preoperative anticancer treatments. The exclusion criteria were as follows: (1) Patients aged < 65 years; (2) Intrahepatic cholangiocarcinoma or mixed-type HCC was confirmed by postoperative pathological examination; (3) Patients have absolute contraindications for liver resection; (4) Severe dysfunction of vital organs; (5) History of any other malignancy.

### 2.2 Data collection

For the included patients, we collected the basic information of the patients at the time of admission in the medical records, including age, sex, overweight (BMI>24), viral hepatitis B, liver cirrhosis, abdominal surgery history, hypertension, diabetes, chronic obstructive pulmonary disease (COPD), tumor size (cm), tumor numbers (single or multiply), tumor location (VII/VIII/IVa or not), alpha-fetoprotein (AFP, ng/L), indocyanine green retention rate at 15 minutes (ICGR15, %), TB (mmol/L), ALB (g/L), aspartate aminotransferase (AST, IU/L), alanine aminotransferase (ALT, IU/L), platelet (PLT, IU/L), white blood count (WBC, 10^9^/L).

In addition, we also collected the data of patients undergoing surgical treatment, postoperative pathological data, and postoperative complications in the medical records. The information includes ASA(I~IV), surgical type(laparoscopic or open), major resection, blood loss(ml), intraoperative transfusion, total pringle time(min), margin distance(mm), cancer embolus, microvascular invasion, capsular invasion, microsatellite nodules, poor differentiation, necrosis (0~4), fibrosis (0~4), overall complications, major complications, liver-specific complications, liver failure, hemorrhage, ascites, biliary leakage, general complications, respiratory complications, respiratory infection, wound infection, pleural effusion, atelectasis respiratory insufficiency, Clavien-Dindo Grade(I~IV) and hospital stay.

All patients were followed up every 1 month for the first 3 months after discharge and every 3 months thereafter. The median follow-up time was 34.5 months. The primary endpoints of the study were overall survival (OS) and disease-free survival (DFS), and the secondary endpoint was complication rate. DFS was defined from the end of surgery to death or recurrence; OS was defined from the end of surgery to death.

### 2.3 Statistical analysis

All patients were randomly assigned into training cohort and validation cohort in a ratio of 3:1. For descriptive statistics of patient clinical characteristics data, the statistical description median [interquartile range (IQR)] was used for continuous variables, and frequency (%) was used for categorical variables. Univariate Cox regression was used to screen the risk factors related to prognosis. Prognostic variables were further selected by least absolute shrinkage and selection operator regression model (LASSO) and multivariate Cox regression to identify the predictors of overall survival (OS) and disease-free survival (DFS). Proportional hazards assumption was used to assessed the Cox regression models. These indicators were then used to construct a predictive nomogram.

A series of validation methods were used to validate the accuracy and discrimination of the nomogram, including AUC, calibration curve and consistency index (C-index). In order to explore clinical application value of the model, decision analysis curve (DCA) was used to calculate net benefit under different thresholds. At the same time, we divided all patients into low-risk and high-risk groups based on each patient’s nomogram score. Log-rank test and Kaplan-Meier (K-M) curve were used to compare survival differences among patients in different groups.

All data analyses were performed using R 4.1.1. and SPSS 25.0. All tests were two-sided, and a P<0.05 was considered statistically significant.

## 3 Result

### 3.1 Baseline characteristics

**Figure 1:**
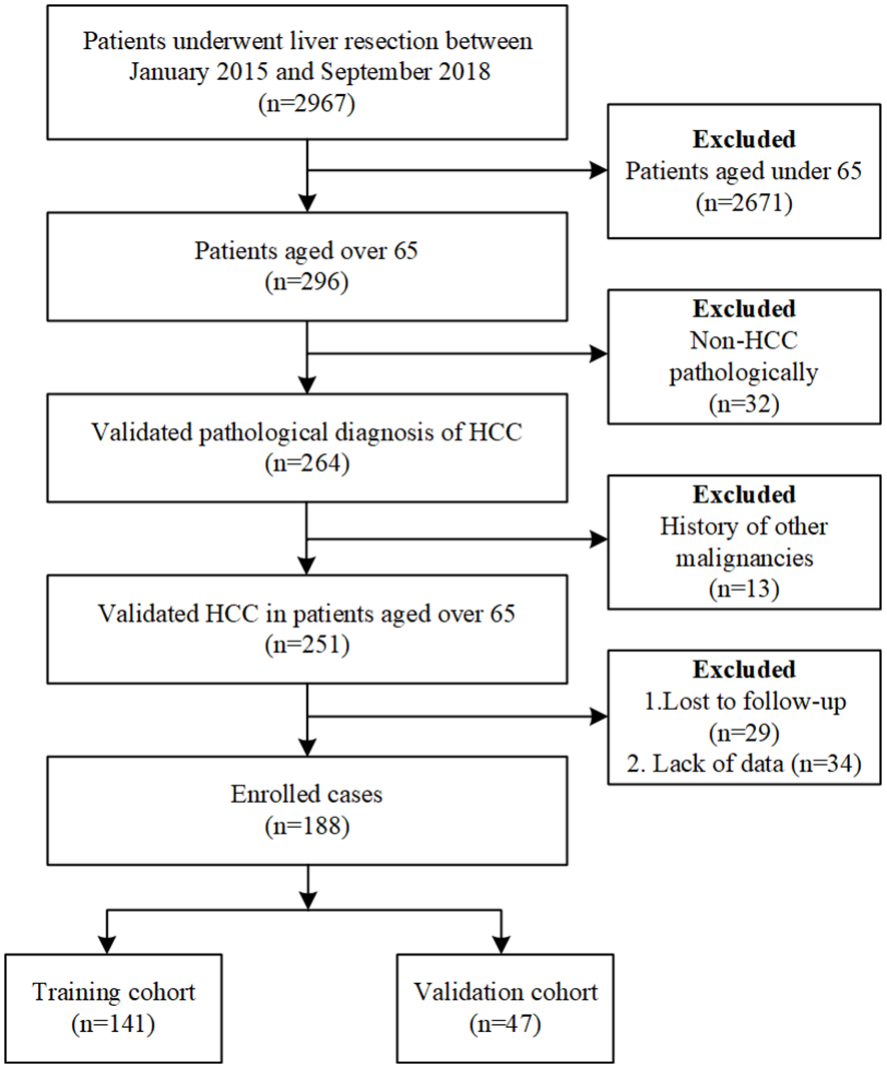
Flow chart of included and excluded patients in the study.

A total of 188 elderly HCC patients who underwent hepatectomy were included in this study, as shown in **Figure 1**. Of these patients, males accounted for 77.66% and the median age was 69 (66,72) years. Among them, 40.43% of the patients were overweight (BMI>24). 39.36% and 41.49% of the patients had a history of hepatitis B and liver cirrhosis respectively. 29.26% of the patients had abdominal surgery history. In terms of common chronic diseases in the elderly, hypertension accounted for 34.57%, diabetes accounted for 17.55%, and COPD accounted for only 3.19% (**Table 1**).

**Table 1:** Characteristics of 188 elderly patients with hepatocellular carcinoma.

Variables	All (N=188)	Training cohort (N=141)	Validation cohort (N=47)	P
Age (years)	69 (66,72)	69 (66, 72)	70 (67, 74)	0.689
Sex (Male%)	146 (77.66%)	108 (76.60%)	38 (80.85%)	0.544
Overweight (BMI>24)	76 (40.43%)	58 (41.13%)	18 (38.30%)	0.731
Viral hepatitis B	74 (39.36%)	53 (37.59%)	21 (44.68%)	0.389
Liver cirrhosis	78 (41.49%)	55 (39.00%)	23 (48.94%)	0.232
Abdominal surgery history	55 (29.26%)	44 (31.21%)	11 (23.40%)	0.309
Hypertension	65 (34.57%)	46 (32.62%)	19 (40.43%)	0.330
Diabetes	33 (17.55%)	22 (15.60%)	11 (23.40%)	0.223
COPD	6 (3.19%)	4 (2.83%)	2 (4.26%)	0.632
Tumor size (cm)	5.65 (4.00, 7.00)	5.50 (4.00, 7.50)	6.00 (4.00, 7.00)	0.915
Tumor numbers (single)	137 (72.87%)	105 (74.47%)	32 (68.09%)	0.394
Tumor location (VII/VIII/IVa)	96 (51.06%)	71 (50.35%)	25 (53.19%)	0.736
AFP (ng/L)	8.23 (3.77, 132.10)	8.03 (3.59, 133.35)	8.28 (4.41, 132.1)	0.736
ICGR15 (%)	6.90 (4.03, 10.50)	6.90 (3.58, 10.50)	7.40 (4.40, 10.50)	0.804
TB (mmol/L)	14.25 (10.53, 18.45)	14.40 (10.65, 18.10)	13.50 (9.60, 19.80)	0.517
ALB (g/L)	41.50 (37.75, 44.45)	41.30 (37.65, 44.30)	42.40 (37.90, 44.50)	0.631
AST (IU/L)	35.00 (25.00, 52.00)	36.00 (26.00, 53.00)	32.00 (24.00, 51.00)	0.278
ALT (IU/L)	31.50 (19.00, 48.75)	33.00 (19.00, 52.00)	31.00 (19.00, 47.00)	0.743
PLT (IU/L)	126.00(86.25,172.00)	122.00 (84.50, 169.50)	144.00 (98.00, 175.00)	0.141
WBC (10^9^/L)	5.60 (4.17, 7.09)	5.45 (4.09, 6.96)	5.98 (4.33, 7.50)	0.328

The median tumor size of patients was 5.65cm (4.00,7.00). The tumors of 72.87% of the patients were single lesions, and the tumors of 51.06% of the patients were located in the posterosuperior segments (VII/VIII/IVa) (**Table 1**). Part of the blood routine, blood biochemical, and serum tumor marker data of the patients are shown in **Table 1**. All data were collected at the time of admission. **Table 2** shows the surgical, pathological, and postoperative information of 188 patients.

**Table 2:** Surgical, pathological and postoperative information of 188 patients.

Variables	All (N=188)	Training cohort (N=141)	Validation cohort (N=47)	P
ASA				0.770
I	13 (6.91%)	10 (7.09%)	3 (6.38%)	
II	139 (73.94%)	103 (73.05%)	36 (76.60%)	
III	35 (18.62%)	27 (19.15%)	8 (17.02%)	
IV	1 (0.53%)	1 (0.71%)	0 (0.00%)	
Surgical type (Laparoscopic: open)	91:97	70:71	21:26	0.555
Major resection	13 (6.91%)	9 (6.38%)	4 (8.51%)	0.868
Blood loss (ml)	300 (150, 400)	300 (150, 400)	300 (200, 400)	0.737
Intraoperative transfusion	21 (11.17%)	14 (9.92%)	7 (14.89%)	0.349
Total pringle time (min)	30 (13, 45)	32 (15, 45)	22 (0, 45)	0.431
Margin distance (mm)	10 (2, 10)	10 (2, 10)	10 (2, 10)	0.538
Cancer embolus	6 (3.19%)	3 (2.13%)	3 (6.38%)	0.338
Microvascular invasion	63 (33.51%)	46 (32.62%)	17 (36.17%)	0.656
Capsular invasion	116 (61.70%)	90 (63.83%)	26 (61.70%)	0.299
Microsatellite nodules	37 (19.68%)	25 (17.73%)	12 (25.53%)	0.244
Poor differentiation	110 (58.51%)	85 (60.28%)	25 (53.19%)	0.393
Necrosis				0.277
0	0 (0.00%)	0 (0.00%)	0 (0.00%)	
1	33 (17.55%)	21 (14.89%)	12 (25.53%)	
2	94 (50.00%)	73 (51.77%)	21 (44.68%)	
3	54 (28.72%)	42 (29.79%)	12 (25.53%)	
4	7 (3.72%)	5 (3.55%)	2 (4.26%)	
Fibrosis				0.102
0	0 (0.00%)	0 (0%)	0 (0.00%)	
1	20 (10.64%)	9 (6.38%)	11 (23.40%)	
2	43 (22.87%)	34 (24.11%)	9 (19.15%)	
3	54 (28.72%)	43 (30.50%)	11 (23.40%)	
4	71 (37.77%)	55 (39.01%)	16 (34.04%)	
Overall complications	149 (79.26%)	111 (78.72%)	38 (80.85%)	0.755
Major complications	5 (2.66%)	3 (2.13%)	2 (4.26%)	0.749
Liver-specific complications	127 (67.55%)	94 (66.67%)	33 (70.21%)	0.653
Liver failure	3 (1.60%)	2 (1.42%)	1 (2.13%)	0.737
Hemorrhage	3 (1.60%)	3 (2.13%)	0 (0.00%)	0.737
Ascites	123 (65.43%)	91 (64.53%)	32 (68.09%)	0.658
Biliary leakage	2 (1.06%)	1 (0.71%)	1 (2.13%)	0.439
General complications	15 (7.98%)	12 (8.51%)	3 (6.38%)	0.877
Respiratory complications	19 (10.11%)	14 (9.92%)	5 (10.64%)	1.000
Respiratory infection	5 (2.66%)	3 (2.13%)	2 (4.26%)	0.794
Wound infection	3 (1.60%)	3 (2.13%)	0 (0.00%)	0.574
Pleural effusion	12 (6.38%)	8 (5.67%)	4 (8.51%)	1.000
Atelectasis	3 (1.60%)	3 (2.13%)	0 (0.00%)	0.574
Respiratory insufficiency	1 (0.53%)	1 (0.71%)	0 (0.00%)	1.000
Clavien-Dindo Grade				0.697
I	123 (65.43%)	92 (65.25%)	31 (65.96%)	
II	21 (11.17%)	16 (11.35%)	5 (10.64%)	
IIIA	0 (0.00%)	0 (0.00%)	0 (0.00%)	
IIIB	2 (1.06%)	1 (0.71%)	1 (2.13%)	
IV	3 (1.60%)	2 (1.42%)	1 (2.13%)	
Hospital stay (days)	7 (5, 8)	7 (5, 8.5)	7 (6, 8)	0.743

In the training cohort, 46 patients died, while in the validation cohort, 15 patients died. The 1-year and 3-year OS of the training cohort was 83.7% and 72.3% of that of the validation cohort, respectively, and the OS of the validation cohort was 85.1% and 74.5%, respectively.

### 3.2 Univariate Cox regression analysis of OS and DFS

In order to initially determine the factors related to the OS and DFS of the elderly patients, univariate Cox regression analysis was performed. The results showed that ALB, viral hepatitis B, TB, PLT, cancer embolus, capsular invasion, major resection, microvascular invasion, blood loss, and overweight (BMI>24) were significantly related to the OS of the patients (**Table 3**). ALB, major resection, capsular invasion, TB, pleural effusion, microvascular invasion, and PLT were significantly related to the DFS of the patients (**Table 3**).

**Table 3:** Univariate Cox regression analysis of OS and DFS in 141 elderly patients with hepatocellular carcinoma after operation.

Variables	HR (95% CI)	P value
OS		
ALB (g/L)	0.01(0.002~0.04)	<0.001
Viral hepatitis B	3.19(1.76~5.79)	<0.001
TB (mmol/L)	2.34(1.49~3.69)	<0.001
PLT (IU/L)	0.47(0.30~0.74)	0.001
Cancer embolus	5.19(1.59~16.97)	0.006
Capsular invasion	2.47(1.22~4.99)	0.011
Major resection	2.98(1.16~7.61)	0.023
Microvascular invasion	1.97(1.09~3.56)	0.026
Blood loss (ml)	1.30(1.02~1.66)	0.035
Overweight (BMI>24)	1.87(1.04~3.38)	0.037
Total pringle time (min)	0.90(0.80~1.01)	0.083
Hospital stay (days)	1.51(0.92~2.48)	0.099
Pleural effusion	2.16(0.76~6.14)	0.149
Hypertension	1.53(0.84~2.78)	0.162
DFS		
ALB (g/L)	0.05(0.01~0.22)	<0.001
Major resection	3.73(1.66~8.37)	0.001
Capsular invasion	2.16(1.24~3.75)	0.006
TB (mmol/L)	1.63(1.11~2.38)	0.012
Pleural effusion	2.08(1.18~6.65)	0.019
Microvascular invasion	1.65(1.01~2.72)	0.046
PLT (IU/L)	0.69(0.47~1.00)	0.050
Cancer embolus	2.84(0.88~9.09)	0.079
Viral hepatitis B	1.53(0.94~2.51)	0.090
Blood loss (ml)	1.18(0.97~1.44)	0.095
ASA	0.46(0.19~1.16)	0.100
Hospital stay (days)	1.39(0.93~2.08)	0.113
Laparoscopic surgery	0.70(0.43~1.13)	0.146
Respiratory complications	1.73(0.82~3.63)	0.148

### 3.3 LASSO regression analysis of OS and DFS

**Figure 2:**
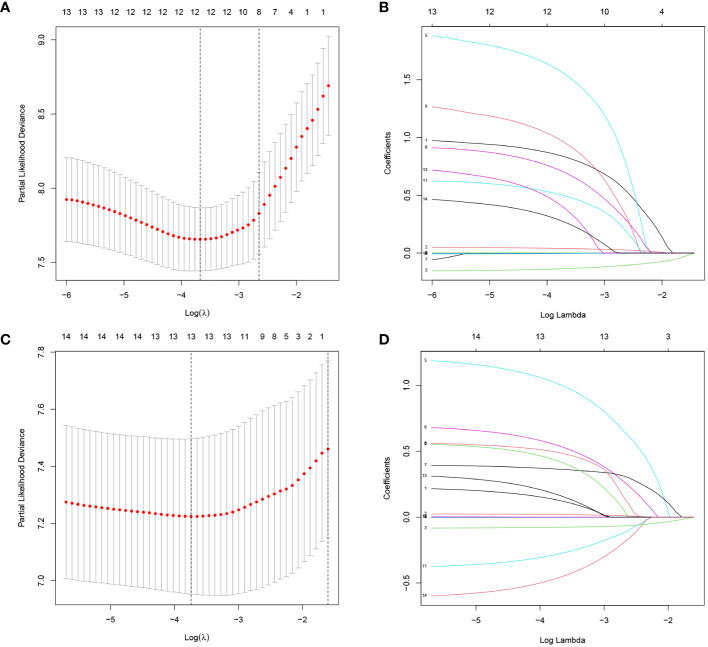
Screening of variables based on Lasso regression. **(A)** OS, **(C)** DFS: The selection process of the optimum value of the parameter λ in the Lasso regression model by cross-validation method. **(B)** OS, **(D)** DFS: The variation characteristics of the coefficient of variables.

The LASSO regression analysis was used to reduce high-dimensional data (14). The features with a P value <0.2 in the univariate Cox regression analysis of OS and DFS were included in the LASSO. The cross-validation was used to determine the most appropriate λ value as optimal parameters (**Figure 2**). The results for OS yield a λ value of 12, and the results for DFS yield a λ value of 13. Indicating that 12 features associated with OS and 13 features associated with DFS included in LASSO were important.

### 3.4 Multivariate Cox regression analysis of OS and DFS

Significant variables output by LASSO regression analysis were included in multivariate Cox regression analysis. The results of multivariate Cox regression analysis showed that ALB, cancer embolus, blood loss, viral hepatitis B, TB, microvascular invasion, overweight (BMI>24), and major resection were independent risk factors for OS in elderly patients (**Table 4**). Major resection, ALB, microvascular invasion, laparoscopic surgery, blood loss, TB, and pleural effusion were independent risk factors for DFS in elderly patients (**Table 4**). The proportional hazards assumption was evaluated and found reasonable for each variable (**Table S1**).

**Table 4:** Multivariate Cox regression analysis of OS and DFS in elderly patients with hepatocellular carcinoma after operation.

Variables	HR (95% CI)	P value
OS		
ALB (g/L)	0.85(0.79~0.91)	<0.001
Cancer embolus	9.03(2.45~33.27)	<0.001
Blood loss (ml)	1.01(1.00~1.02)	<0.001
Viral hepatitis B	2.86(1.52~5.40)	0.001
TB (mmol/L)	1.05(1.01~1.09)	0.007
Microvascular invasion	2.49(1.25~4.96)	0.009
Overweight (BMI>24)	2.27(1.17~4.41)	0.016
Major resection	3.36(1.17~9.67)	0.024
DFS		
Major resection	4.56(1.92~10.81)	<0.001
ALB (g/L)	0.91(0.86~0.96)	0.001
Microvascular invasion	2.02(1.17~3.47)	0.012
Laparoscopic surgery	0.54(0.32~0.89)	0.016
Blood loss (ml)	1.01(1.00~1.02)	0.020
TB (mmol/L)	1.03(1.00~1.06)	0.039
Pleural effusion	2.68(1.02~7.04)	0.046

### 3.5 Establishment of the nomogram and accuracy evaluation

**Figure 3:**
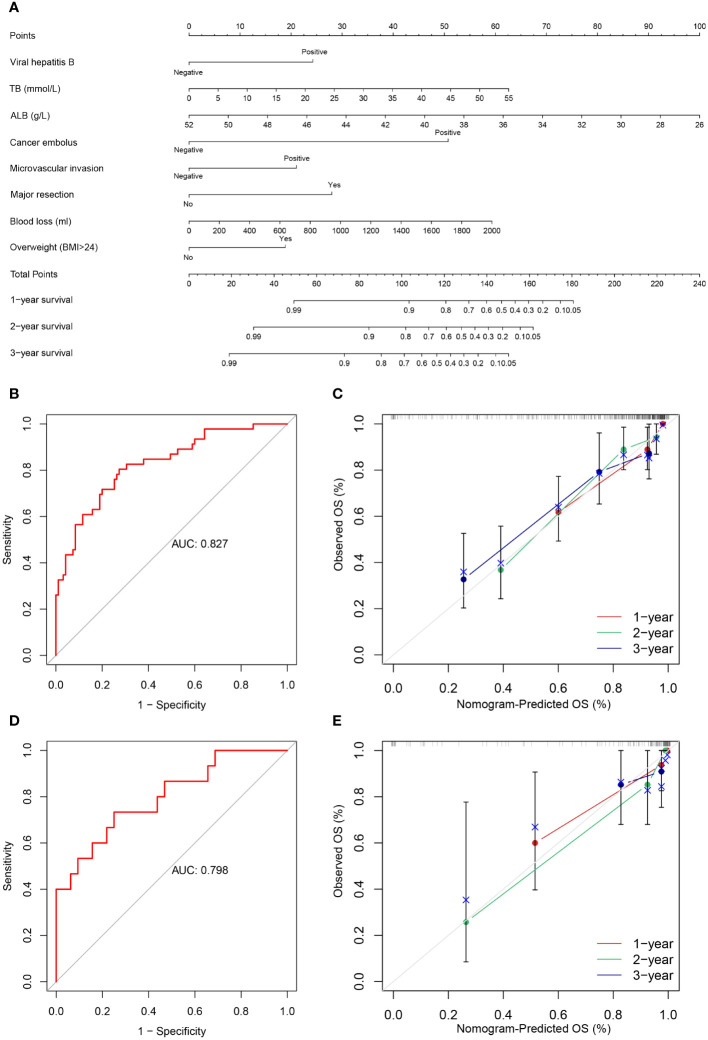
**(A)** Nomogram prediction model and prediction OS curve; **(B)** AUC for predicting OS in training cohort; **(C)** Calibration curves of 1-, 2-, and 3-year OS in training cohort; **(D)** AUC for predicting OS in validation cohort; **(E)** Calibration curves of 1-, 2-, and 3-year OS in the validation cohort.

**Figure 4:**
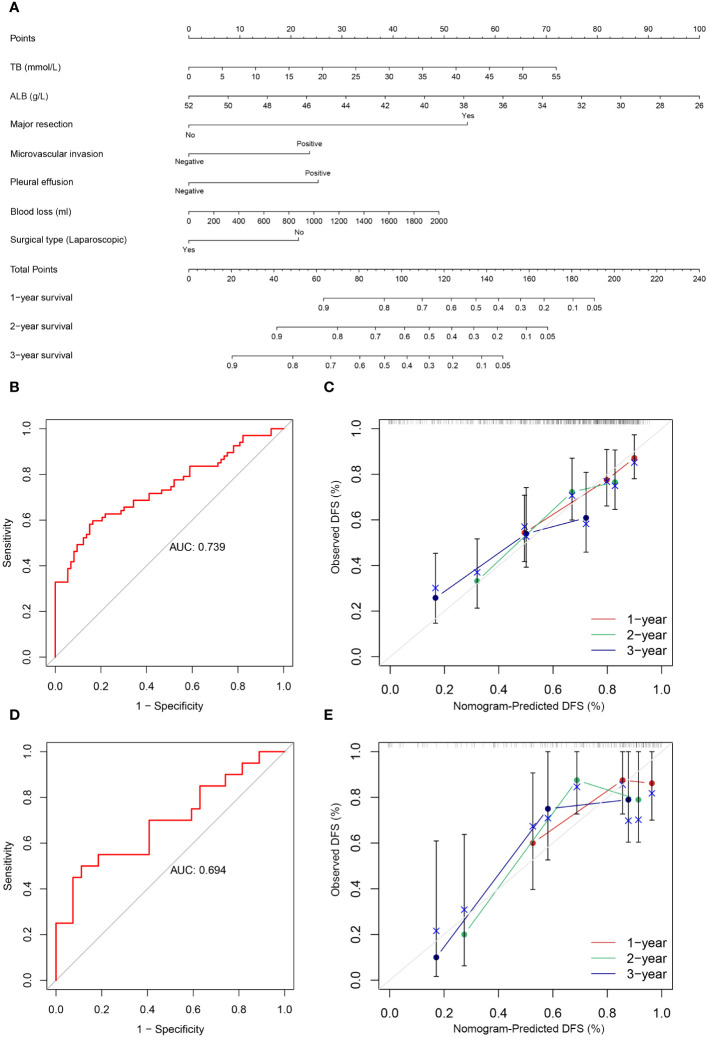
**(A)** Nomogram prediction model and prediction DFS curve; **(B)** AUC for predicting DFS in training cohort; **(C)** Calibration curves of 1-, 2-, and 3-year DFS in training cohort; **(D)** AUC for predicting DFS in validation cohort; **(E)** Calibration curves of 1-, 2-, and 3-year DFS in the validation cohort.

The following factors were used to construct the nomogram (**Figures 3**, **4**). 1-, 2-, and 3-year OS and DFS in elderly patients were predicted by the nomogram. We used ROC curves to verify the predictive value of these independent prognostic factors. As shown in **Figures 3** and **4**, the area under the curve (AUC value) of the eight independent prognostic factors for the prediction of OS was 0.827. The AUC value of 6 independent prognostic factors for the prediction of DFS was 0.739. In the validation cohort, the AUC values were 0.798 and 0.694, respectively. This suggests that these factors have good predictive value for OS and DFS. Subsequently, calibration curves were depicted, and the results showed that the predicted values of the prediction models were generally consistent with the actual observed values (**Figures 3**, **4**).

### 3.6 Clinical application of the nomogram

**Figure 5:**
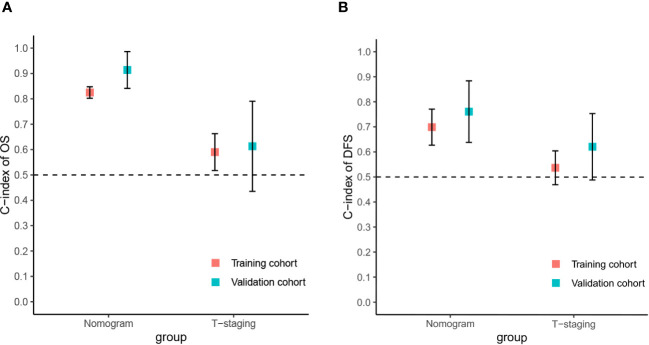
The C-index of the nomograms and T-staging. **(A)** The C-index of the OS nomogram and T-staging; **(B)** The C-index of the DFS nomogram and T-staging.

**Figure 6:**
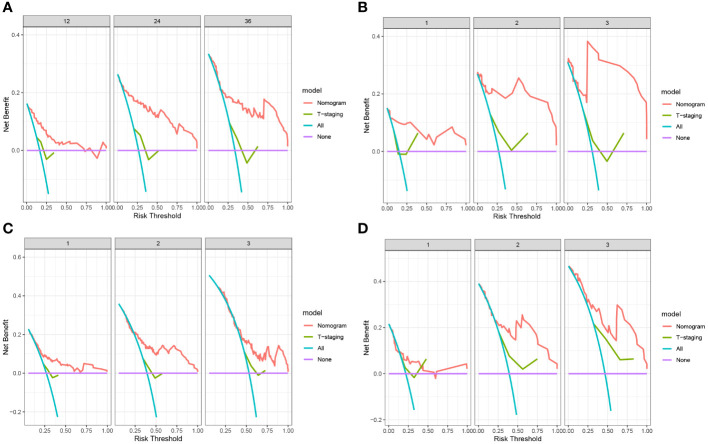
**(A)** DCA of OS in training cohort; **(B)** DCA of OS in validation cohort; **(C)** DCA of DFS in training cohort; **(D)** DCA of DFS in validation cohort.

The predictive value of the constructed nomogram was compared with the 8th edition American Joint Committee on Cancer (AJCC) T-staging system in terms of clinical practicability. The results are shown in **Figure 5**. In the training cohort, the C-index of the nomogram for OS and DFS was 0.825 and 0.699, respectively, which was significantly higher than that of the T-staging (OS: 0.590; DFS: 0.537). Similarly, in the validation cohort, the C-index of the nomogram for OS (0.914) and DFS (0.761) was also significantly higher than that of the T-staging (OS: 0.613; DFS: 0.621). Additionally, DCA suggested that the nomogram had better predictive power than the T-staging (**Figure 6**). Overall, the nomogram models exhibited superior performance compared to the T-staging.

**Figure 7:**
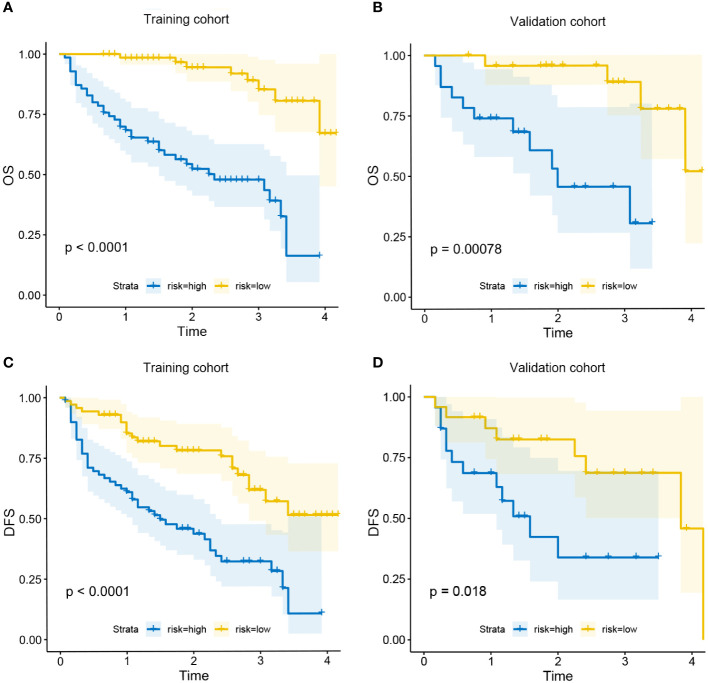
Kaplan-Meier curves for OS in the low-risk and high-risk groups in training cohort **(A)** and validation cohort **(B)**; Kaplan-Meier curves for DFS in the low-risk and high-risk groups in training cohort **(C)** and validation cohort **(D)**.

All patients were assigned to the high-risk group or the low-risk group based on their nomogram scores. In both the training and validation cohorts, patients in the low-risk group exhibited significantly higher survival rates and lower recurrence rates than those in the high-risk group (**Figure 7**).

## 4 Discussion

### 4.1 The significance of nomogram

At present, the prognosis prediction model of malignant tumor patients has been widely used. In recent years, the nomogram related to HCC has been gradually developed, which has played a very active role in the diagnosis, treatment, and prognosis prediction of HCC. For example, Li et al. constructed a nomogram to predict the risk of liver nodule malignant transformation into HCC, which is helpful for the early diagnosis of HCC (15). Lin et al. established a simplified model to help guide decisions about prophylactic transarterial chemoembolization after hepatectomy for patients with HCC (16). Wang et al. developed a nomogram to predict recurrence in patients with early-stage HCC (17). However, the prognosis of HCC patients is closely related to age. In addition, the incidence of HCC is highest in the elderly population, whose postoperative prognostic factors are different from other age groups. For example, the underlying health conditions of elderly patients and the higher incidence of postoperative complications will affect the prognosis of patients. Currently, there is still a lack of predictive models for recurrence and survival after liver resection in elderly patients, so we constructed a nomogram to predict the prognosis of such elderly patients.

Unlike earlier prognostic indicators such as BCLC and Child–Pugh grade (18), the nomogram we developed calculates the survival rate and recurrence rate for each patient rather than simply categorizing patients into different risk groups. This approach reduces the impact of heterogeneity, thereby aiding clinicians in making individualized treatment decisions for elderly HCC patients and establishing a foundation for managing high-risk patients in clinical practice.

### 4.2 Summary of main risk factors

Our study collected the clinical information of elderly HCC patients over 65 years old who underwent liver resection in our hospital. After univariate analysis, LASSO regression analysis, and multivariate Cox analysis, it was found that ALB, cancer embolus, blood loss, viral hepatitis B, TB, microvascular invasion, overweight (BMI>24), and major resection were closely related to the OS of this group of people. Major resection, ALB, microvascular invasion, laparoscopic surgery, blood loss, TB, and pleural effusion are closely related to the DFS of this group. Among them, ALB, blood loss, TB, microvascular invasion, and major resection are related to both DFS and OS. Previous studies have shown that these factors are associated with the prognosis of HCC.

#### 4.2.1 Discussion of risk factors associated with basic characteristics

Many previous studies have shown that a serum ALB level of <35g/L is a risk factor for prognosis of HCC. As for the mechanism, ALB has been shown to inhibit HCC growth, migration and invasion (19, 20). In a recent investigation by Zeng et al. (21), focusing on young patients with HCC, it was elucidated that a lower ALB level correlated with increased recurrence after liver resection. ALB and TB are important parameters to access liver functional estimation in the Child-Pugh score. But in recent years, more and more studies have used the Albumin-Bilirubin (ALBI) grade to evaluate the liver reserve function and prognosis prediction ability of HCC patients (22–24). Our study discovered that ALB and TB levels affect the prognosis of elderly HCC patients as independent predictive factors, which is consistent with previous studies.

For elderly patients, there is still insufficient evidence of whether overweight increases the risk of poor prognosis after liver resection. Currently, there is only limited evidence that obesity itself does not affect the prognosis of postoperative patients with HCV-related HCC (25). Another study shows that overweight and obese patients with cirrhosis have an increased morbidity rate after hepatectomy (26). However, we found that being overweight may be a risk factor for OS in elderly HCC patients undergoing hepatectomy.

HBV has long been considered an independent risk factor associated with poor prognosis in HCC. High HBV replication rate and related non-resolving inflammation are major risk factors of postoperative recurrence, and antiviral treatment can effectively prolong postoperative survival (27). But our study only found that HBV was associated with OS in elderly patients, not with DFS.

#### 4.2.2 Discussion of risk factors associated with surgical resection

The effect of whether to perform major hepatectomy on prognosis is still controversial. On the one hand, extended liver resection can benefit those patients with large and locally advanced HCC (28), but at the same time, it will increase the burden on the residual liver and increase the risk of liver failure (29). However, we found that major resection is a risk factor in older patients.

Suh et al. (30) revealed that intraoperative blood loss ≥ 700 mL were risk factors for tumor recurrence after surgical resection for HCC, consistent with the findings of our study. Large-volume blood loss may impede the immune reaction against tumor cells and induce hypoxic ischemia, thereby increasing the likelihood of tumor recurrence (31).

As for the choice of surgical method, many studies recommend laparoscopic surgery for elderly patients (32). Our study further confirmed that laparoscopic surgery is sufficiently safe, has no significant impact on OS, and can reduce the risk of recurrence. On the one hand, laparoscopic resection is significantly associated with less blood loss, wider resection margins, shorter hospital stays, and lower morbidity (33). On the other hand, smaller tumors are more inclined to be eligible for laparoscopic resection. Challenges persist in laparoscopic approaches, particularly with lesions located in the posterosuperior segments, large and recurrent tumors, and in cases of advanced cirrhosis (34).

#### 4.2.3 Discussion of risk factors associated with pathological characteristics

As is known to us, tumor embolism is seen most commonly in metastatic renal cell carcinoma; hepatocellular carcinoma; and carcinomas of the breast, stomach, and prostate (35). For tumor embolism caused by HCC, studies have shown that it is associated with poor prognosis of HCC (36, 37). This is also consistent with our conclusion. For microvascular invasion, it is also an established risk factor for HCC (38), which not only can affect OS but also DFS for elderly HCC patients, due to its association with microscopic residual metastatic disease after resection (39). Microvascular invasion and tumor embolism are associated with circulating tumor cells (CTCs). CTCs are independent significant risk factors for HCC recurrence and can be identified as biomarkers for diagnosis, prognostication, and therapeutic monitoring.

#### 4.2.4 Discussion of risk factors associated with postoperative complications

Previous studies have shown that complications after hepatectomy in HCC patients will affect their prognosis (40). For the elderly, their reserve capacity is low, and many comorbidities, such as cardiovascular disease, diabetes, and respiratory system disease, etc., make postoperative rehabilitation and treatment difficult (32, 41). Therefore, the incidence of postoperative complications is higher than that of patients in other age groups. Our data showed that postoperative pleural effusion was an independent risk factor for DFS. Due to the limited number of patients with various complications in our included patients, we did not find that the occurrence of other postoperative complications was related to the prognosis of the patients.

### 4.3 Strengths and limitations

Overall, the clinical features we used to construct the nomogram after statistical screening were reported in previous studies. It is suggested that these factors may be related to the OS and DFS of patients. However, in terms of these clinical features, no studies have systematically analyzed them in elderly HCC patients undergoing hepatectomy and determined their impact on prognosis. We initially confirmed the validity of the nomogram for OS and DFS prediction in elderly HCC patients who underwent hepatectomy, and found that it exhibited superior predictive power compared to T-staging, as suggested by DCA. This nomogram can effectively identify patients at high risk of death and recurrence. Therefore, the model we developed is an intuitive clinical tool with good predictive performance, assisting physicians in making rational treatment decisions for elderly HCC patients.

However, this study still has some limitations. First of all, we constructed the nomogram based on the clinical data of West China Hospital of Sichuan University. This does not necessarily represent other countries and regions. Secondly, as a retrospective analysis, this study inevitably has information bias and selection bias, which may affect the conclusion to a certain extent. Third, the nomogram we constructed has only been internally verified, and more clinical data and multi-center studies are still needed for external verification to further prove the effectiveness of the nomogram.

## 5 Conclusion

We have built and internally validated a nomogram to identify the risk factors of overall survival and recurrence in elderly HCC patients who underwent hepatectomy. Although the nomograms have exhibited better prediction for OS and DFS, further multi-center studies and external verification are still needed.

## Data availability statement

The original contributions presented in the study are included in the article/**Supplementary Material**. Further inquiries can be directed to the corresponding authors.

## Ethics statement

The studies involving humans were approved by Biomedical Ethics Review Committee of West China Hospital of Sichuan University. The studies were conducted in accordance with the local legislation and institutional requirements. The ethics committee/institutional review board waived the requirement of written informed consent for participation from the participants or the participants’ legal guardians/next of kin because 1.The medical records used in this study were obtained from previous clinical records. 2.In this study, the risk to patients is no greater than the minimum risk. 3.Exemption from informed consent will not have adverse effects on the rights and health of the subjects. 4.The privacy and personal identity information of the subjects are protected. 5.This study does not utilize medical records that patients have explicitly refused to use in the past. 6.This study does not involve personal privacy and commercial interests.

## Author contributions

YT: Data curation, Methodology, Validation, Writing – original draft. YW: Formal analysis, Investigation, Resources, Visualization, Writing – original draft. NW: Investigation, Methodology, Software, Visualization, Writing – original draft. YL: Data curation, Resources, Supervision, Writing – review & editing. GL: Project administration, Resources, Software, Writing – review & editing. BL: Conceptualization, Funding acquisition, Resources, Supervision, Writing – review & editing.
