# Supplementary material for: Development and validation of nomogram to predict overall survival and disease-free survival after surgical resection in elderly patients with hepatocellular carcinoma
Source: Front Oncol. 2024 May 24;14:1395740. doi: 10.3389/fonc.2024.1395740 (PMC11157056; doi:10.3389/fonc.2024.1395740)
Supplement: Supplementary file 1 [file Table_1.docx]

**Table S1. The proportional hazards assumption for variables.**

| **Variables** | **χ_­_^2^** | **P value** |
| --- | --- | --- |
| **OS** |  |  |
| Viral hepatitis B | 3.526 | 0.06 |
| TB (mmol/L) | 0.151 | 0.70 |
| ALB (g/L) | 2.706 | 0.10 |
| Cancer embolus | 0.860 | 0.35 |
| Major resection | 0.086 | 0.77 |
| Microvascular invasion | 0.766 | 0.38 |
| Blood loss (ml) | 2.537 | 0.11 |
| Overweight (BMI>24) | 1.078 | 0.30 |
| **DFS** |  |  |
| TB (mmol/L) | 0.222 | 0.64 |
| ALB (g/L) | 0.488 | 0.48 |
| Major resection | 1.673 | 0.20 |
| Microvascular invasion | 0.374 | 0.54 |
| Pleural effusion | 0.924 | 0.34 |
| Blood loss (ml) | 0.598 | 0.44 |
| Surgical type | 0.027 | 0.87 |
